# T2-mapping and T1-mapping detect myocardial involvement inTako-Tsubo cardiomyopathy: a preliminary experience

**DOI:** 10.1186/1532-429X-17-S1-P354

**Published:** 2015-02-03

**Authors:** Emmanuelle Vermes, Lauriane Pericart, Julien Pucheux, Anne Delhommais, Daniel Alison, Olivier Genee

**Affiliations:** 1 Cardiac Imaging, CHU Trousseau, Tours, France

## Background

T2- and T1-mapping are novel CMR techniques allowing objective tissue characterization. These techniques have been shown to be superior to dark blood imaging in NSTEMI patients in detecting ischaemic area at risk and acute oedema. These methods have not been assessed in Tako-Tsubo cardiomyopathy (TC).The aim of the study was to assess myocardial involvement using T2- and T1-mapping in Tako-Tsubo cardiomyopathy (TC).

## Methods

Nine patients with TC and 15 controls were prospectively enrolled. Cardiovascular magnetic resonance (CMR) was performed a mean 2.8 days after the onset of symptoms and after a mean 4.6 month follow-up. CMR was applied using T2-mapping, pre and post contrast T1-mapping (MOLLI) and LGE sequences. Segmental and global T1 values have been measured before and after contrast administration. Wall motion abnormality (WMA) was assessed.

## Results

All patients were female, had positive troponin (6±9µg/l) and medium and/or apical ballooning associated with moderate LV dysfunction (EF 44±7%). On admission, compared with controls, TC patients had significantly higher T2 values (65±6 ms vs 50±4 ms, p<0.0001). Myocardial T2 was significantly higher in segments with WMA compared to normokinetic segments (67±12 ms vs 61.5±8 ms, p=0.003). Compared with controls, TC patients had significantly higher pre contrast T1 values (1115±92 versus 1016±89, p<0.0001) and significantly lower post contrast T1 values (428±24 ms vs 466±19 ms, p=0.02).Pre contrast T1 values were significantly higher in segments with WMA compared to normal segments (1126±95 vs 1089±85, p=0.016). Post contrast T1 values were not significantly different in abnormal segments compared to normal segments (421±56 vs 431±50, p=0.15). No patients had LGE (assessed visually). At follow-up: all had a complete LV recovery (EF: 67±4%) without significant WMA. Mean T2 and pre contrast T1 values decreased significantly at follow up (53±6ms vs 65±8ms, p=0.001 and 1016± 76 vs 1115 ± 80, p=0.001 respectively). No differences were observed regarding post contrast global T1 values.

## Conclusions

In TC patients, T2-mapping and pre contrast T1-mapping allow identification of reversible myocardial injury. Post contrast T1 mapping does not provide additional information.

## Funding

No funding.

